# Comparative Evaluation of Postoperative Sensitivity Using Three Different Tooth-Colored Restorative Materials in Non-carious Cervical Lesions: A Split-Mouth Design In Vivo Study

**DOI:** 10.7759/cureus.27861

**Published:** 2022-08-10

**Authors:** Radhika Gupta, Aditya Patel, Pradnya Nikhade, Manoj Chandak, Rutuja Rajnekar, Meghna Dugar

**Affiliations:** 1 Conservative Dentistry and Endodontics, Sharad Pawar Dental College and Hospital, Datta Meghe Institute of Medical Sciences, Wardha, IND

**Keywords:** flowable composites, resin modified glass ionomer cement, split-mouth design, schiff cold air sensitivity scale, zirconomer

## Abstract

Introduction

Three different tooth-colored restorative materials were evaluated and compared for postoperative sensitivity using a split-mouth design. This is a double-blinded clinical trial assessed for a one-month follow-up period in patients with non-carious cervical lesions (NCCLs).

Materials and Methods

A total of 60 NCCLs in 20 participants were considered in this split-mouth design study and randomly divided into three different groups: Flowable composite (n = 20), resin-modified glass ionomer cement (RMGIC) (n = 20), and Zirconomer® (n = 20). The restorations were evaluated for postoperative sensitivity at baseline (BL-day 0), three, seven, and 21 days using the Schiff cold air sensitivity scale. Data were analyzed using IBM SPSS Statistics for Windows, Version 23.0 (Released 2015; IBM Corp., Armonk, New York, United States) using a post hoc test for postoperative sensitivity and one-way Anova to analyze all the groups together at the time interval of three, seven, and 21 days.

Results

In Group 1 (flowable composites) and Group 3 (Zirconomer), a statistically significant difference in terms of reduced postoperative sensitivity was seen after three and seven days. However, a significant reduction in postoperative sensitivity was seen after three, seven, and 21 days in Group 2 (RMGIC).

Conclusion

In this study, RMGIC showed reduced postoperative sensitivity after restoring NCCLs compared to Zirconomer and flowable composites. Compared with flowable composites and Zirconomer, RMGIC showed better clinical performance in terms of less postoperative sensitivity after restoring NCCLs.

## Introduction

The problem of tooth wear is now progressively becoming significant to dental professionals. Disease processes other than dental caries such as pathological loss of tooth structure exist that are referred to as loss of tooth surface [1]. Non-carious cervical lesions (NCCLs) are caused by processes that result in the loss of hard tissue or restorative material in the cervical third of the teeth [2]. These cervical lesions are categorized into three types: (i) Abrasion, which refers to the pathological wear of tooth substance induced by repetitive frictional processes caused by external mechanical causes, such as tooth brushing, (ii) Erosion, which refers to the dissolution of tooth structure owing to acid damage, either intrinsic or extrinsic, and (iii) abfraction, which refers to pathological tooth loss induced by a concentration of stresses in the cervical area due to high nonaxial loading pressures [3-6].

Whenever hypersensitivity, cosmetic issues, or visible flaws occurs due to NCCLs, they should be restored with appropriate restorative material; otherwise, it may lead to the initiation of caries, pulpal infections, or even displacement of the tooth [7]. Restoring NCCLs, however, is a challenging task because it doesn't provide any retention form due to its saucer or wedge-shaped cavity; also, the formation of sclerotic dentine is not suitable for adhesion on the tooth surface and margins of the cervical area, which is frequently placed sub-gingivally obstructing the filling access, thereby making the moisture control difficult [8,9].

The motive for restoring the NCCLs is to replace the lost tooth structure as well as to occlude the open dentinal tubules. Most of the NCCLs have mixed cavity margins positioned on the enamel, dentin, or cementum. However, restoring this form of the cavity can be questionable with restorative materials that bond likewise to enamel and dentin. The retention of the restorative materials employed in the cervical area may be negatively impacted by several structural factors. Therefore, adhesive materials such as resin-based composites (RBCs) and glass ionomer cement (GICs) are preferred choices for the restoration of NCCLs [10].

Self-cured GIC has many clinical uses in NCCLs. For the restoration of lesions in the cervical third of the tooth, GIC has been widely employed, as it releases fluoride, prevents caries, and most importantly binds chemically to both enamels as well as dentin. However, while GICs are used regularly because they have lower mechanical properties than RBCs, they are comparatively unesthetic. To compensate for these shortcomings of GICs, resin-modified GIC (RMGIC) was introduced with added resin components that are both self-curing and light-curing. This addition has shown higher moisture resistance to contact and desiccation, equal fluoride release, increased early compressive plus flexural strength, as well as better mechanical characteristics [10,11].

RBCs are more acceptable aesthetically than GICs. However, they are associated with postoperative sensitivity, polymerization shrinkage, microleakage, recurrent caries, and marginal discoloration. Adhesive resin composites can involve several steps during restoration based on the selected material. Flowable composite resins were introduced in the late 1990s that are now being commonly used due to their better handling property [12]. In combination with a transparent matrix, flowable composites are considered more efficient to restore NCCLs [13]. It is highly advisable to use a flowable composite to restore NCCLs for their lower modulus of elasticity [14]. Reinforced with zirconia and ceramic fillers, a newer variety of GICs, Zirconomer® (Shofu Inc., Japan), was introduced to provide better compressive as well as flexure strengths. It has also been considered a high-strength restorative material, which showed less occlusal wear and fast setting reaction. The mechanical properties of Zirconomer are comparable to GIC as well as amalgam [13]. Studies about the comparative assessment of the microleakage and post-operative sensitivity of Zirconomer are sparse in the literature.

Hypersensitivity is considered to be the most important factor while restoring NCCLs. It is defined as the pain caused by a non-significant stimulus. According to current research, it has been found that with an increase in age, susceptibility to hypersensitivity increases due to the presence of NCCLs [14]. The condition when the gum recedes and leads to the exposure of a cervical zone of the tooth to the oral environment is called gingival recession [14]. Gingival recession leads to loss of hard structure and sensitivity in the area involved. Postoperative sensitivity is often observed after the restoration of NCCLs. So far, adequate data about the comparison of postoperative sensitivity in NCCLs using RMGIC, Zirconomer, and flowable composites is scanty. There was a need to conduct clinical research to identify which restorative material amongst the most recommended ones is most efficient in reducing postoperative sensitivity in patients with NCCLs.

Dental clinical research typically employs split-mouth designs, in which a mouth is split into two or more experimental halves that are randomly assigned to various treatments. Split-mouth designs are utilized in this study since it has been considered the most effective tool for carrying out such clinical research. It is an illustration of a site-level randomization technique in which two treatments are assigned at random locations in each of the two compartments of the mouth. The benefit of this design is that it significantly reduces the inter-individual variability from the estimates of the treatment impact. It is employed because each participant serves as their control, requiring fewer people to achieve the same level of study power as the parallel group. Additionally, each participant receives all interventions, thus it's useful for figuring out preferences [15].

Consequently, the goal of this split-mouth, double-blinded clinical trial was to assess and compare the postoperative sensitivity of three different tooth-colored restorative materials for restoring NCCLs. The null hypothesis assumes that all the three restorative materials would perform equally well in the restoration of NCCLs over one month.

## Materials and methods

Study design

This interventional study has been conducted in the Department of Conservative Dentistry and Endodontics, Sharad Pawar Dental College, Sawangi (Meghe), Wardha, India. The Institutional Ethics Committee of Datta Meghe Institute of Medical Sciences (Deemed to be a University) approved the study protocol (Ref.No.DMIMS (DU)/IEC/2020-21/264). The study enlisted the participation of patients who visited the outpatient department. All patients were informed about the benefits, risks, and alternative treatment options before enrolment. It was mandatory to complete an informed consent form by all the patients who participated in this clinical trial. The split-mouth approach was used in this prospective, double-blind (volunteers and examiners) investigation with a one-month follow-up period examining the clinical performance of flowable composite resins, RMGIC, and Zirconomer after the restoration of NCCLs in anterior and posterior teeth. The study trial was registered at the Indian Council of Medical Research (ICMR)-National Institute of Medical Sciences Clinical Trial Registry of India (CTRI/2022/02/040682).

Participants and lesion selection

The present clinical trial has been double-blinded. By using computer-aided simple random sampling, restorative materials were assigned to the patients. The numbers were randomly selected, with each representing a different restorative material. Three different tooth-colored restorative materials were selected in the study: flowable composites, RMGIC, and Zirconomer. Participants were recruited and treated at Sharad Pawar Dental College and Hospital from October to March, 2022. The recruitment process included 27 volunteers who satisfied the inclusion criteria. Finally, 20 individuals were encompassed in the trial, along with three sets of teeth. For each patient, a pair of teeth were matched by considering three teeth from different quadrants with similar positions. A week before restoration, periodontal scaling was completed.

In the department, we had diagnosed 60 NCCLs cases in 20 patients (at least three in each) regardless of etiology. Based on the case history of the patients, it was found that the toothbrushing method followed by patients was cross brushing of the arches. In diagnoses, it is been attended that the size of the lesion would not be over one-third of the crown length of the tooth. Width and depth measurements of lesions were not considered in the study. In the study, the common properties of the lesions were: the occlusal margins of the lesions were at the enamel tissues or occlusal margin and the gingival margins of the lesions were at the dentine tissues. None of the lesions had reached the pulpal tissue. Following are the inclusion and exclusion criteria for the study (Table 1).

**Table 1:** Inclusion and exclusion criteria for the study DMFT: decayed, missing, and filled teeth

Inclusion criteria	Exclusion criteria
Twenty participants aged 25 to 40 were included in the study	Patients with medical conditions, advanced periodontitis, high-risk caries, parafunctional habits, i.e., bruxism, xerostomia, shattered or cracked teeth, and expecting or breastfeeding moms were excluded from the study
Non-smoker and good oral hygiene	Patients who have mobile or fixed prosthetic restorations near their teeth are restored
Patients with healthy periodontal status	Patients with any systemic condition, as well as a positive history of antibiotic and analgesic usage in the week leading up to the treatment
Patients with tooth sensitivity	The study eliminated teeth with caries involving the cervical area, crack teeth, pulpal and peri-radicular involvement, mobile teeth, and restorations contacting the buccal sides
Patients having aesthetic concerns were included in the study	DMFT index of more than 12, poor dental hygiene, orthodontic equipment, and severe bruxism are all factors to consider
Low caries index	
Teeth selection	
Wedge or saucer-shaped lesions	
Vital teeth	
Periodontitis, peri-radicular lesions, traumatic occlusion, bruxism, and wear aspects are non-existent	

Restorative procedure

Detailed case history of patients was recorded. All restorations were conducted by only one trained and experienced specialist. A total of 60 NCCLs were randomly divided into three groups (n=20). Before the implantation of the restorations, all of the individuals were required to complete their oral prophylaxis. A pear-shaped, coarse diamond bur (EX-41; Mani Inc., Tochigi, Japan) was used to minimally and intermittently roughen the surface of the lesions, which would be restored by flowable composites under high speed and water cooling. No retention grooves or bevels were created. The appropriate shade was selected using a shade selection guide. Saliva suction device, cotton rollers, and cheek retractors were used to isolate the operative area. Following the recommendations by the manufacturers, the NCCLs in Group 1 (Flowable composites) were restored with flowable composites. The lesions to be restored with Group 2 (RMGIC) and Group 3 (Zirconomer) were prepared without using any rotary instruments following American Dental Association (ADA) requirements for enamel and dentin adhesive material that doesn’t permit placement of bevel. A rubber dam was used to isolate the lesions and according to the manufacturer's instructions, the restoration was completed.

Following that, tungsten carbide bur for finishing and Sof-Lex™ disc (3M Company, Saint Paul, Minnesota, United States) for polishing was used to complete the restoration under water spray. Proper brushing technique was advised to all the participants using a soft toothbrush and toothpaste every day following the modified bass method. Participants were recalled at the end of three, seven, and 21 days for the assessment of restorations done. The restorations were assessed for postoperative sensitivity by the Schiff cold air sensitivity scale. The investigator was blinded for the use of restorative material in the samples employed for the study.

Criteria used for clinical evaluation

One specialist with experience conducted a clinical examination and was blinded to all evaluations at baseline (day 0), three days, seven days, and 21 days. The Schiff cold air sensitivity scale [16,17] was used for assessing the restorations for postoperative sensitivity; the tooth was isolated using cotton rolls, following that a few drops of extremely cold water were delivered through a syringe to the tooth, and thus sensitivity was evaluated using this scale. This scale was taken into account to evaluate the participant's reaction to the cold stimuli (Table 2).

**Table 2:** The Schiff cold air sensitivity scale

Score	Interpretation
Score 0	Subject does not respond to air stimulus
Score 1	Subject responds to air stimulus but does not request discontinuation of stimulus
Score 2	Subject responds to air stimulus and requests discontinuation or moves from the stimulus
Score 3	Subject responds to air stimulus, considers stimulus to be painful, and requests discontinuation of the stimulus

The average of the results obtained from two baseline-designated study teeth were used to compute each subject's score.

Data analysis

Data were analyzed using IBM SPSS Statistics for Windows, Version 23.0 (Released 2015; IBM Corp., Armonk, New York, United States) using a post hoc test for postoperative sensitivity to analyze all the groups together at the time interval of three, seven, and 21 days respectively.

## Results

The preoperative sensitivity of all three groups was recorded before commencement of treatment, i.e., at baseline (day 0), against which future comparisons were made after three, seven, and 21 days. The baseline values for the flowable composite, RMGIC, and Zirconomer were 1.2±0.41, 1.2±0.76, and 1.1±0.55, respectively (Table 3).

**Table 3:** Comparison of the flowable composite, RMGIC, and Zirconomer groups at each timepoint RMGIC: resin-modified glass ionomer cement

	Flowable Composite				RMGIC				Zirconomer			
	Day 0 Baseline	three days	7 days	21 days	Day 0 Baseline	3 days	7 days	21 days	Days 0 baseline	3 days	7 days	21 days
Mean	1.2	0.7	0.8	1	1.2	0.1	0.2	0.2	1.1	0.5	0.6	0.8
Standard Deviation	0.41	0.47	0.41	0	0.76	0.30	0.41	0.41	0.55	0.51	0.50	0.41
Standard Error	0.09	0.10	0.09	0	0.17	0.06	0.09	0.09	0.12	0.11	0.11	0.09
Minimum	1	0	0	1	0	0	0	0	0	0	0	0
Maximum	2	1	1	1	2	1	1	1	2	1	1	1

The mean difference in terms of reduced postoperative sensitivity for Group 1 (Flowable composite) compared to day 0 (baseline) value after three and seven days was 0.5±0.51 with a p-value of 0.001 and 0.4± 0.68 with a p-value of 0.006, respectively, which shows the statistically significant result. Similarly, the mean difference after 21 days was 0.2±0.41 with a p-value of 0.33, which was not statistically significant.

The mean difference in terms of reduced postoperative sensitivity for Group 3 (Zirconomer) compared to day 0 (baseline) value after three and seven days was 0.6±0.68 with a p-value of 0.001 and 0.5± 0.60 with a p-value of 0.01, respectively, which shows the statistically significant result. Similarly, the mean difference after 21 days was 0.3±0.47 with a p-value of 0.23, which was not statistically significant.

The mean difference in postoperative sensitivity reduction for Group 2 (RMGIC) compared to day 0 (baseline) value after three, seven, and 21 days was 1.1 ± 0.85, 1 ± 0.79, 1 ±0.64, respectively, with a p-value of 0.001, which shows the statistically significant result. Group 1 and Group 3 showed a statistically significant difference in postoperative sensitivity reduction after three and seven days. However, Group 2 showed a statistically significant difference after three, seven, and 21 days, respectively. When all the three groups were compared, Group 2 showed the most statistically significant difference in postoperative sensitivity reduction (Table 4).

**Table 4:** Comparison of values of postoperative sensitivity using post hoc test in flowable composite, RMGIC, and Zirconomer groups RMGIC: resin-modified glass ionomer cement; HSD: honestly significant difference

Group	Timeline	Mean Difference	Tukey HSD p-value
Flowable composite group	Baseline (day 0) -3 days	0.5±0.51	0.001
	Baseline (day 0) -7 days	0.4± 0.68	0.006
	Baseline (day 0) -21 days	0.2±0.41	0.33
RMGIC group	Baseline (day 0) -3 days	1.1 ± 0.85	0.001
	Baseline (day 0) -7 days	1 ± 0.79	0.001
	Baseline (day 0) -21 days	1 ±0.64	0.001
Zirconomer group	Baseline (day 0) -3 days	0.6 ± 0.68	0.001
	Baseline (day 0) -7 days	0.5 ± 0.60	0.01
	Baseline (day 0) -21 days	0.3 ± 0.47	0.23

In intergroup evaluation, the baseline to three-, seven-, and 21-day comparison showed statistically significant results with flowable composite-RMGIC and RMGIC-Zirconomer when compared (Table 5).

**Table 5:** Comparison of values of postoperative sensitivity using post hoc test in flowable composite, RMGIC, and Zirconomer groups RMGIC: resin-modified glass ionomer cement; HSD: honestly significant difference

Timeline	Timeline	Tukey HSD p-value
Baseline (day 0) -3 days	Flowable composite– RMGIC	0.02
	Flowable composite – Zirconomer	0.64
	RMGIC – Zirconomer	0.06
Baseline (day 0) -7 days	Flowable composite – RMGIC	0.02
	Flowable composite – Zirconomer	0.88
	RMGIC – Zirconomer	0.06
Baseline (day 0) -21 days	Flowable composite – RMGIC	0.001
	Flowable composite – Zirconomer	0.79
	RMGIC – Zirconomer	0.001

## Discussion

NCCL is a common occurrence and presents the dentist with significant restorative difficulties. Resin-based composites, traditional GICs, RMGICs, and compomers are the general categories used to classify dental aesthetic Class V restorative materials [18]. The amount of missing tooth structure to be replaced depends upon the aesthetic concerns, remaining dentinal thickness, along with buccolingual and occlusal-gingival dimensions. All these factors have an impact on the selection of an appropriate restorative material for NCCLs. For the restoration of NCCLs, composite resins together with dentin bonding agents had been regarded as the most efficient over time [19,20]. In this present study, 60 teeth with NCCLs were handled by a solo operator to minimize operator bias and restored using three different tooth-colored restorative materials, flowable composites, RMGIC, and Zirconomer.

Postoperative sensitivity after treating NCCLs is a common problem encountered in a clinical scenario. Increased postoperative sensitivity from the patient's perspective would be an undesirable result [21]. In a study by Arhun et al., 33% of 456 restored teeth had a significant prevalence regarding postoperative sensitivity [22]. However, as the time period increases, because of using self etch primer, the smear layer is dissolved, incorporating it into the mixture of collagen fibers and resin monomers, which results in the smear layer becoming an integral part of the hybrid layer resulting in less or no postoperative sensitivity. Casselli reported that among 104 composite resin restorations, 47% had postoperative sensitivity [23]. The variations in the composite resins and adhesive systems that were used, along with the methodology of the studies, may have contributed to the discrepancies that were discovered. Therefore, the whole purpose of the current study was to find out which restorative material results in the lowest postoperative sensitivity while treating NCCLs.

Filtek™ Z250 XT flowable composite (3M Company, Saint Paul, Minnesota, United States) was utilized in this present study. Excellent aesthetics, increased wear resistance, and excellent polishing are the advantages provided by its special nanotechnology. The stresses induced by polymerization shrinkage are often absorbed by these flowable composite resins. Given this, flowable composite resins are recommended as a cavity wall liner and thus, completing the restoration with a material having high moduli of elasticity and a large number of filler particles. The modulus of elasticity for flowable composite is 11,000 MPa [24]. Another material is Zirconomer, a white amalgam a new class of glass ionomer restorative material that has high strength due to the presence of zirconia fillers. It has the combined property of amalgam exhibiting high strength, durability, and bonding, fluoride-releasing properties of GIC [13].

RMGIC induced the minimum stresses in the cervical area of the tooth, both with and without an occlusal restoration. Due to the low modulus of elasticity, the material may get deformed or flexed, absorbing the produced stresses and reducing the resulting stresses in the tooth [25]. This is consistent with the findings of Van Meerbeek et al., which verified the association between enhanced clinical outcomes with a lower modulus of elasticity [26]. The detrimental effects of tooth flexure are the most crucial component of the NCCLs restoration. Materials with low elastic moduli are recommended for restoration because they are more flexible and can withstand occlusal forces that concentrate in the cervical regions. Low-modulus materials are preferred because high-modulus materials cannot flex when the tooth structure is deformed under load, which can result in displacement from the cavity [27]. Thus, in this present study, when flowable composite resins, RMGIC, and Zirconomer were compared to evaluate postoperative sensitivity in NCCLs, it was seen that RMGIC due to its low moduli of elasticity (10,000 MPa) was the best choice of material to restore NCCLs over Zirconomer and flowable composites.

When a comparison was done in this study between flowable composites, RMGIC, and Zirconomer groups in each timepoint and each test, significant differences in terms of decreased postoperative sensitivity for RMGIC were seen after three, seven, and 21 days. Whereas, for Zirconomer and flowable composites, decreased postoperative sensitivity was seen after three and seven days and increased sensitivity after 21 days of restoration respectively. As the study was a split-mouth design, it was easy to unswervingly compare the reaction to the three dissimilar restorative materials in each patient [28].

Dhanapal and Sureshbabu compared the post-operative sensitivity of two different flowable composite restorations in NCCLs using cold water, cold ice stick, and air blast. A visual analog scale (VAS) was used to record the sensitivity scores at baseline, two days, one week, and four weeks after the therapy, and it was concluded that in terms of postoperative sensitivity reduction there was no significant difference in each test and in each timepoint [29]. Stojanac et al. evaluated the clinical performance of three dissimilar tooth-colored restorative materials in NCCLs and complete regression of postoperative sensitivity was seen after 24 months [14].

Perdigao and colleagues [30] concluded that increased sensitivity at the start of treatment results from gingival recession and exposure to the root surface of the tooth, which occurs right away after inserting a restoration or just after it has been finished and polished. Sensitivity that developed right away after the restoration was put in place could be the result of excess material that came into touch with soft tissues or mechanical damage done to the gingiva during finishing and polishing. If the surface of the repair is well-polished, without steps or uneven areas, gingivitis will be reversible [11]. Previous research has shown that several parameters, including isolation methods, curing modes, enamel beveling, placement methods, as well as further enamel etching, substantially reduce postoperative sensitivity [29].

The limitations of this study are small sample size and lesser duration for evaluation of postoperative sensitivity. Hence, longer-duration studies with increased sample sizes need to be considered to further investigate the ideal material for the restoration of NCCLs.

## Conclusions

Within the constraints of this research, after a one-month evaluation period, flowable composites and Zirconomer showed a statistically significant difference in postoperative sensitivity reduction after three and seven days while a reduction in postoperative sensitivity was seen after three, seven, and 21 days in RMGIC. RMGIC can be used to restore NCCLs satisfactorily by employing the right restorative techniques. The exclusion of etiological variables and good oral hygiene are additional significant aspects to increase the longevity and quality of restorations.
